# Eating as an autistic adult: An exploratory qualitative study

**DOI:** 10.1371/journal.pone.0221937

**Published:** 2019-08-29

**Authors:** Emma Kinnaird, Caroline Norton, Caroline Pimblett, Catherine Stewart, Kate Tchanturia

**Affiliations:** 1 Institute of Psychiatry, Psychology and Neuroscience, Kings College London, London, United Kingdom; 2 South London and Maudsley NHS Foundation Trust, London, United Kingdom; 3 Illia State University, Tbilisi, Georgia; University of Colorado Denver School of Medicine, UNITED STATES

## Abstract

**Background:**

Although eating difficulties are known to be common in children on the autism spectrum, there is a lack of research on whether these behaviours persist or change into adulthood. Emerging evidence suggests that autistic adults may experience higher levels of disordered eating than the general population, indicating the impact of autism on eating in this adult population warrants further exploration.

**Method:**

This study interviewed 12 autistic adults about their eating habits, with a focus on the continuing or changing presence of behaviours often seen in autistic children such as sensory sensitivity or a preference for routines. Interviews were transcribed and analysed using thematic analysis.

**Results:**

Overall, participants suggested that autism did continue to impact their eating into adulthood, particularly in the areas of sensory sensitivity, medical difficulties, executive functioning difficulties, and rigidity, but that they had learned to adapt so that these issues no longer represented a problem. However, a minority of participants did feel that their autism had a negative effect on their eating, particularly those diagnosed with eating disorders. Additionally, eating behaviours associated with autism were identified as potentially contributing to having an unhealthy body weight.

**Conclusions:**

Certain traits associated with autism, such as cognitive rigidity and sensory sensitivity, could potentially continue to influence the eating behaviours of autistic adults. These traits are typically experienced as differences which can be adapted around and managed, rather than specific problems. However, these traits can potentially contribute to difficulties such as disordered eating and weight gain, and the implications of these should be explored by future research.

## Introduction

The link between selective eating and autism in children is well-established in the research literature [1–3]. Children on the autism spectrum are more likely to have a restricted diet, refusing more foods and eating a more limited food repertoire than their typically developing peers [4–6]. This selectivity appears to be motivated by a number of factors. Studies suggest that children who exhibit higher levels of sensory sensitivity are more likely to refuse foods on the basis of sensory qualities, including texture, temperature, taste and smell [7]. Atypical sensory processing is common in autism [8], and selective eating in autism is associated with sensory sensitivity [1, 4, 9, 10]. The preference for routine, and behavioural inflexibility, associated with autism may also cause problems with eating, making children reluctant to try new foods or leading to the development of specific mealtime routines [4, 9, 11]. Eating problems may also be driven by physical difficulties associated with autism, such as motor problems (including chewing, or using utensils) or gastrointestinal symptoms [2, 12, 13]. Furthermore, the diets of children on the autistic spectrum may be deliberately restricted by parents- for example, gluten- or casein-free diets- with the goal of improving gastro-intestinal symptoms, or changing behaviour [14].

The nutritional impact of these restricted eating behaviours is less clear. Whilst children on the spectrum eat a more limited diet, the findings of research suggest that this does not necessarily lead to overall insufficient nutritional intake [15–17]. Although these children may be at risk of under-consuming specific nutrients, including calcium, fibre, folic acid, Vitamins A, D, and E [17–20], research suggests that this population may also over-consume certain food types. For example, there is evidence that children on the autistic spectrum may be at greater risk of having their weight classified in the overweight or obese range, potentially due to eating a limited diet of carbohydrate rich foods [21, 22].

However, there is comparatively less research on whether these eating behaviours associated with autism extend into adulthood [23]. (Please note that identity first language (e.g. autistic adults) will be used to refer to this population. A study found that this style is generally preferred by autistic adults, and identity first language was used by all participants in their interviews [24]). Investigating eating in autism beyond young childhood, one study found that food selectivity in autism, defined as only eating certain foods, was less pronounced in older children and adolescents compared to younger children, suggesting that this phenomenon may change as children get older [6]. However, research does suggest that some of these eating behaviours do persist in autistic adults. Autistic adolescents and adults are more likely to be reluctant to try new foods and exhibit more food sensitivities compared to their neurotypical peers [25]. Similar behaviours were found in a study examining eating problems in autistic adults with intellectual disabilities [26]. This reflects research suggesting that autistic adults similarly experience atypical sensory processing, including impacting the ability to taste [27–30]. A recent questionnaire designed to measure eating problems in autistic adults based on a previous literature review (primarily using research on children) and author clinical experience identified a number of other difficulties that may be experienced by this population, including motor control, environmental aspects surrounding mealtimes including social pressures, difficulty judging hunger or satiety, routine-focused behaviours, and executive functioning problems [23, 31].

The issue of whether eating problems persist into adulthood for autistic people is significant for two key reasons. Firstly, interventions or support developed to support people on the autistic spectrum with eating are primarily targeted at young children and their parents, and may not be appropriate for autistic adults. This is especially important as the food experiences of autistic adults are likely to be different to those of children: for example, adults are more likely to be expected to choose and prepare their own food. This would make problems such as the refusal of food provided by other people less relevant, but may cause other difficulties, such as around the executive functioning skills required to plan, purchase and cook meals [32]. Secondly, atypical eating behaviours may have a negative impact on both physical and mental health: research suggests that autistic adults may be at a higher risk of being overweight [33]. Furthermore, research in the eating disorder (ED) field suggests that there may be an association between autism and EDs [34]. Consequently, the aim of this study was to use qualitative methods to explore whether autism may impact eating for some autistic individuals in adulthood, and how far this is perceived by these individuals as a problem.

## Method

### Participant recruitment

Participants who had previously responded to an online study entitled “Problematic Eating on the Autism Spectrum” and gave permission to be contacted regarding future research were invited to participate in the study. The online study had previously been advertised on social media (Twitter), and was open to both autistic and neurotypical adults with and without a history of eating problems. Participants were eligible if they were over 18, and if they self-reported an autism diagnosis. Participants were excluded if they were not sufficiently fluent in English to participate in an interview.

### Procedure

The study received ethical approval from London-City and East Research Ethics Committee and South London (18/ LO/0050). A total of 50 individuals were contacted, and a researcher explained the purpose of the study. 38 individuals did not respond or declined to take part, leaving a total sample of 12. All participants provided written informed consent. Interviews were conducted by over video conferencing (e.g. Skype), over the phone, or over instant messenger, depending on participant preference. The participants had no prior relationship with the interviewer but were made aware that the interviewer was neurotypical rather than autistic. Interviews were semi-structured, using a topic guide exploring eating behaviours and aspects of eating known to be atypical in autism, such as sensory sensitivity and selectivity. Participants were first asked to self-report demographic information, including their age, current weight and height, any history of an ED, and if they were experiencing any medical conditions impacting their eating. Participants were then asked questions exploring the role of autism in their eating, such as “Do you think that your autism affects your eating?” and “What would you like medical professionals to know about eating and autism?”. Participants with a current ED were additionally asked more targeted questions about their ED, including “How well do you feel that current understandings of your ED apply to you and your experiences?” and “How do you understanding ED recovery in the context of your autism?”. The interviewer was free to ask follow-up questions in response to topics raised by the participant. Each interview lasted between 20–45 minutes and was audio-recorded, with field notes made during each interview. No repeat interviews were carried out, and transcripts were not returned to participants. As this sample represented all eligible individuals who gave consent to participate from the initial online study, data saturation was not discussed.

### Analysis

The study is presented in line with Consolidated criteria for reporting qualitative studies (COREQ) guidelines [35]. Data was analysed using thematic analysis [36]. Transcripts were read and reread by authors EK and KT to ensure familiarisation. An initial set of codes were then produced by EK based on the content of the transcripts, evaluated by KT, and applied to the data using NVivo 11 software. Coded data was then analysed to identify potential themes. These themes were then reviewed by EK and KT, evaluating how well they captured the coded data, and how far they reflected the entire data set. Responses to interview questions were highly heterogenous, reflecting the diversity of food experiences and attitudes endorsed by participants. However, within the responses, some unifying themes were identified: Autism and Eating, Impact, and Coping and Adapting. These were divided into sub-themes and are presented below.

## Results

### Participants

The final sample consisted of 12 participants: 2 men, 8 women, and 2 participants who identified as non-binary. Demographic information is summarised in Table 1. Participants were a mean age of 38.50 years (*SD* = 13.87), with a mean body mass index (BMI) of 25.61 (*SD* = 8.10). 2 participants did not report their exact weight and height, and instead reported their BMI category (e.g. underweight). 4 participants had a BMI defined as underweight, 2 had a BMI in the normal range, 3 had a BMI defined in the overweight range, and 3 had a BMI defined as obese. 6 participants self-reported experiencing an ED in their lifetime (*n* = 4 anorexia nervosa, AN; *n* = 1 bulimia nervosa; BN, *n* = 1 binge eating disorder; BED).

**Table 1:** Participant demographic characteristics.

Participant	Gender	Age (years)	BMI Category	Age of autism diagnosis (years)	Medical conditions	Eating disorder history
1	Male	23	Overweight	4	Excoriation disorder, trichotillomania, anxiety	Binge eating disorder (current, 3 year duration)
2	Female	46	Normal weight	35		Anorexia nervosa (not formally diagnosed, recovered)
3	Female	41	Normal weight	37	Gluten intolerant	
4	Non-binary	39	Obese	38	Gastrointestinal problems	
5	Female	49	Overweight	48	Egg allergy, migraines	
6	Female	38	Obese	37	Anxiety, obsessive-compulsive disorder, cleft palate	
7	Male	71	Obese	49		
8	Female	49	Overweight	41	Lactose intolerant, food allergies	
9	Female	19	Underweight	15	Anxiety, dysthymic disorder	Bulimia nervosa (recovered)
10	Non-binary	31	Underweight	28	Bipolar disorder, post-traumatic stress disorder, obsessive-compulsive disorder	Anorexia nervosa (current, 4 year duration)
11	Female	34	Underweight	28		Anorexia nervosa (current, 24 year duration)
12	Female	22	Underweight	20		Anorexia nervosa (current, 1 year duration)

### Unifying theme: Autism and eating

While not all participants described having difficulties with eating, they did feel that autism had some kind of impact on their eating. All participants described a degree of selectivity around their eating and food choices: this included avoiding certain foods and eating from only a specific range of food or meals. Autism was also felt to impact their eating in other ways, including leading to difficulties with aspects such as cooking and eating in communal environments. A number of factors associated with participants’ autism were found to contribute towards these behaviours, which are presented below as sub-themes.

#### Sub-theme: Medical issues

A number of participants described having medical problems which affected their eating, typically through motivating them to avoid certain foods or environments. The most common of these were food allergies, leading participants to avoid specific foods. Similarly, one participant who experienced migraines described avoiding certain foods to avoid triggering the condition. Two participants experienced gastrointestinal problems, which again led them to alter their diets by eliminating certain foods, or eating more of foods with a beneficial effect. A further two participants had difficulties with physical coordination, which led them to avoid communal eating environments due to difficulty using cutlery, or fear of social embarrassment. One participant felt that their mental health conditions and associated medication led them to eat less due to stomach discomfort, and one participant avoided communal, loud eating environments due to medical hearing problems making the experience unpleasant.

#### Sub-theme: Sensitivity

Participants described avoiding certain foods, or in one case seeking out specific foods, in order to manage their sensory input. For some, this was avoiding certain foods due to hypersensitivities to aspects like taste, texture, smell, and temperature. While some participants described this in terms of dislikes- “if the texture is off, that food can be just fantastic and I'll still loathe it” (Participant 4), for others this sensitivity was so extreme it caused pain, gagging, or a “meltdown” (Participant 9). While most participants described aversion to specific sensory experiences, such as certain textures, one participant actively used food to increase their sensory stimulation by actively seeking out strong flavours.

Hypersensitivity to certain sensory stimuli also motivated participants to avoid or alter their eating environments. A common experience was avoiding loud environments due to hypersensitivity to noise, which often led to participants avoiding communal eating areas such as restaurants or school dining halls:

“I’d rather not eat in a loud restaurant or dining hall, or just anywhere where I can’t talk to people next to me or just have some peace and quiet… if I’m in an environment where there’s lots of background noise, I find it hard to filter out the background noise. And I think, because I’ve always been good at music, I’m very sensitive to variations in sound” (Participant 2).

These environmental sensitivities meant that participants required a degree of control around their eating environments. Where this kind of control was not available, this sometimes led to avoiding eating in that environment completely:

“I don’t like the smell sometimes that forks and knives have on them. So I will insist on clean cutlery, and if it smells peculiar I will send it back and get clean ones. And in fact sometimes mugs out of the dishwasher have that smell in them as well, and I don’t know what that smell is but it’s pungent. And so if it doesn’t smell just right I won’t eat out of it or on it” (Participant 5).

Participants additionally felt that their eating behaviours were influenced by a difficulty in identifying internal sensations, or interoception- specifically, problems identifying if they were hungry or full. This was viewed by one participant as being closely related to stress levels, and was felt to contribute to under-eating- “it’s the immediate feeling of being full. You know, two bites and you’re full, that’s it. Even if you were a bit hungry, two bites and you’re done” (Participant 3).

#### Sub-theme: Executive functioning

Difficulties with executive functioning also impacted some participants’ eating habits. Problems with aspects such as planning and memory made it difficult to acquire food, with participants describing issues with food shopping, cooking and food preparation, and ordering food. At worst, this led to not eating at all:

“Planning and doing things in the right order can be a big barrier to accessing food. for example, I don't leave my room unless I have a visual mental plan of exactly what I'm going to get, where it is, and how I'm going to get it (often with a backup plan or two so that I don't panic if my first plan is disturbed), and if I don't come up with a satisfactory plan I just don't go at all. Doing things in the right order can be difficult if anything isn't according to plan, like if I drop something or someone tries to talk to me—it's as if once I start executing a plan I enter a sort of autopilot mode, and the system gets lost if it's bumped off course. Oftentimes when that happens I quickly give up and pick something at random to get out as quickly as possible, and that can result in accidentally picking something sensory-bad” (Participant 9).

Consequently, accessing food was viewed by some as requiring a large amount of mental energy and preparation. When participants were distracted or preoccupied by other things, such as stress, this limited the amount of mental energy they could devote to food and eating, again leading to not eating. This appeared to be linked to difficulties with interoception, or having the executive capacity to mentally register the sensation of hunger: “literally forgetting to eat because I just get too busy to, I don’t know, recognise the signals” (Participant 3).

#### Sub-theme: Rigidity and routines

As well as avoiding certain foods, another key behaviour described by participants was that of eating similar foods repeatedly (described by Participant 10 as “samefooding”), or forming a specific routine around eating. This appeared to be related to the mental tendency towards rigidity and routines associated with autism: “you’re quite rigid, you’re quite kind of control orientated, you’re quite kind of perfectionist” (Participant 10).

For a number of participants, food was viewed as a key aspect of life that they could control. This led to repetitive and routine-based eating habits, including a preference for familiar foods and an aversion to trying new things. However, the importance of these routines varied across participants: some described their routines as a preference which could be broken without distress, whereas others were more rigid in their behaviours. For one participant, this rigidity was in part motivated by a fixation on information and numbers. They described finding comfort in their BMI always being at the exact centre of the “healthy” spectrum, and always reading food labels: “I think I’m in control of everything simply because I’m in control of the facts, and I know exactly why I’m doing what I’m doing” (Participant 3).

Rigid thinking patterns also contributed towards eating habits, in particular categorical thinking styles creating aversions towards certain foods. Several participants described having one bad experience with a specific food- for example it causing food poisoning- and not eating that food again: “The milk was sour at school once, and I wouldn’t drink milk for a long time after” (Participant 7). For a small number of participants, this rigidity manifested as a compulsion towards meal completion, regardless of hunger. Alternatively, other participants felt their rigid thinking styles led them to restrict or avoid food. Trying to break these routines or rigid thought patterns was seen as requiring mental effort, “[It is] easier if I don’t have to think about something new to eat. If I know something is good, and that, you know, I’ll feel ok after eating it, then I’m likely to eat it again” (Participant 8).

### Unifying theme: Impact

Although all participants felt that autism influenced their eating to some degree, the impact they felt this had on their lives varied significantly across the sample. The majority of participants did not see their eating behaviours as a problem, but rather a part of their lives which could be managed. However, the participants who had experienced diagnosed EDs did identify the influence of autism on their eating as a significant issue. All participants interviewed in the sample felt that they were still able to get their nutritional needs from the food that they ate, or that they were able to manage any deficiencies with supplements.

#### Sub-theme: Social

Participants identified their behaviours around food as impacting how they behaved in social situations involving eating. This was particularly pronounced around communal eating spaces: firstly, an aversion to certain environments—such as the noise associated with communal spaces like restaurants- prevented or made participants reluctant to attend social events in these locations. Difficulties around eating in communal spaces was related to participants’ abilities to manage their routines and preferences around food, which was made more difficult in social situations. Eating with other people meant that they could not control or avoid experiences or behaviours that they found aversive: for example, if they found overeating distasteful they could not prevent other people overeating, leading to feelings of awkwardness or isolation:

“How do people eat like that? I don’t get it. But I don’t want to eat like that so you know, I don’t feel inspired. I feel a bit repulsed actually, just sit back and observe in grotesque curiosity I suppose” (Participant 3).

Similarly, eating with other people meant that individuals could not always control or select the food being eaten, particularly in the case of family meals eaten at home. Where the food was not palatable or was particularly sensory aversive, participants described eating separately to their family.

As well as being highly aware and sensitive to the behaviours of people around them, some participants were concerned that the people around them would be similarly aware of their own behaviours. These described feeling self-conscious or embarrassed around their own eating habits or difficulties in front of others, leading them to eat alone or only with close acquaintances. For one participant, this aversion to eating in social situations was related to viewing eating food as a purely functional requirement, rather than a pleasurable experience:

“When given the choice, I always prefer to eat alone rather than with people. Eating is not about "hanging out", it's about "putting food in my belly so it stops complaining". There's an element of social anxiety to that, what if I spill food on my shirt… I am self-conscious about the things I am because they're things *I* would be self-conscious about if someone did them around me” (Participant 4).

However, the majority of other participants described still finding enjoyment in eating with other people. Participants generally described preferring, and enjoying, eating with smaller social groups in quieter settings, and so did not experience avoiding busier social environments such as restaurants as a problem.

#### Sub-theme: Weight and eating disorders

Only 2 participants in the sample reported having a BMI in the defined “normal” weight range: all other participants were classified as being in either the under- (*n* = 4) or the overweight/obese (*n* = 6) range. All participants with a BMI in the underweight range either had a current ED diagnosis, or a history of EDs. The overweight participants did not identify their weight as a problem: only one participant, who had previously been overweight but lost weight, saw being overweight as a significant problem in their life. Apart from this participant and individuals with diagnosed EDs, no other participant felt that their eating behaviours caused any physical or health problems.

Both individuals in both the under- and overweight ranges exhibited similar underlying behaviours, but with different effects. For example, both groups described experiencing rigidity around their eating. For those who were underweight this rigidity appeared to contribute towards restriction, or eating only low calorie foods, leading to weight loss. For those who were overweight, this rigidity and repetitiveness tended to be focused around high calorie foods, leading to weight gain:

“I think that sometimes I find that I’ve gotten into a rut with the same thing over and over, and if its something like- something high calorie like chocolate for instance, I struggled with that. Because I started eating it at a certain time of day and then I kept doing that for a long time and I was gaining weight and I noticed that I was constipated and I had to stop that habit. But it’s very easy for me to get into a habit” (Participant 8).

Similarly, both groups described rigid thinking styles that alternatively contributed to under- or overeating. For some, previous experiences had led them to develop a strong compulsion towards completing meals, eating quickly, or over-eating, which they now found difficult to break. Where participants were exposed to certain behaviours or models around eating or body image around childhood, they found these persisted into adulthood and were difficult to break:

“If you’re not taught how to eat well, and you know the sort of behaviours you see as a child is emotional comfort eating, I guarantee you that’s what my mum does. Then that’s the sort of thing you’re exposed to, you know, having a bowl of cereal sized for six people before you go to bed sort of thing, or you know, asking for a bowl of ice cream and you get the entire tub before you go to bed, just because somebody’s upset you you eat a candy bar. You know, that sort of thing, if that’s what you’ve been exposed to, which I was, then that, the sort of patterns that you assume” (Participant 3).

This participant further suggested that the rigid thinking styles associated with autism may make autistic people particularly vulnerable to these kind of environmental influences, especially in childhood:

“We’re all so literal, and all these messages that are out there and all these skinny girls on Instagram- now it’s the bodybuilders on Instagram, you know, the fitness freaks on Instagram… I think these things are all around us, I think we’ve got to think about them very clearly and carefully. Particularly when we’re thinking about how literal autistic people take these messages” (Participant 3).

These experiences therefore led to the formation of certain attitudes or behaviours around food which, combined with the rigidity associated with autism, proved difficult to challenge. For some individuals, this led to under- or overeating, to the degree that their body weight was classified medically as unhealthy, or the development of EDs:

“Autistic people often misuse “black and white thinking”, and I know that affects what and when I eat at home, because if I go to get a snack and my mom says it's too sugary or it's saved for something/someone else, I don't get a different snack, I just don't eat. I think that sort of thing contributed to my eating disorder at the start” (Participant 9).

Across the sample, the participants who identified autism as having the biggest impact on their eating were typically those diagnosed with EDs, although the aspects of autism they felt influenced their eating (such as rigidity) were highly similar to those without EDs. Out of the 12 participants interviewed, 6 reported having experienced an ED in their lifetime: 4 participants had experienced AN, 1 had been treated for BN, and 1 was undergoing treatment for BED. The motivating behaviours underlying their symptoms were highly similar, and strongly overlap, with those seen with those contributing to under- and overeating, and subsequent weight loss or gain. For example, the participant with BED identified a strong compulsive element to their eating leading to overeating:

“I definitely have a tendency towards completion that will cause me to finish a meal that I am not necessarily hungry to finish, and that may be one thing about it. There’s definitely a compulsive element to my eating a lot of times. I also think that, you know, there’s an impulsive element to my eating, and they both sort of come together to, you know, create binge episodes when they do occur” (Participant 1).

Notably, participants with EDs did not typically describe their ED symptoms in terms of emotions, or as strongly influenced by body image. Rather, they strongly related their EDs to the impact of autism, including the role of thinking styles, or viewed it in a functional manner- for example, fulfilling their need for control. The participant with BED explicitly stated that they did not experience the negative emotions around food traditionally associated with binging: rather than binging to cope with negative emotions, they instead found their binging was impulsive and triggered by having access to food (Participant 1).

### Unifying theme: Coping and adapting

The majority of participants described having difficulties with eating during their childhood. As they got older, they felt that they, and the people around them, had consciously worked to manage the influence of autism on their eating to the extent it no longer represented a problem: although all participants felt autism still impacted their eating, they were able to manage and adapt around this impact. Consequently, most participants did not perceive the influence of autism on eating as difficult or problematic.

#### Sub-theme: Progression from childhood

Participants generally described becoming more flexible around food as they became older, with one participant suggesting that their eating became more adventurous with “maturity and self-reflection” (Participant 1). Whilst some participants viewed this as happening gradually, without necessarily requiring conscious effort, others described this as an active process:

“For a long time I would eat only foods I recognized. I worked pretty hard to overcome that… and I have learned to like the experience of trying a new thing, even if it may not be what I know… they've improved because I worked at them” (Participant 4).

Specific changes included making an effort to try new foods, and therefore gradually reducing the associated anxiety associated with unfamiliar foods. Some participants felt that their eating improved from childhood due to support from their family in this process: one participant described how their parents used routines to help introduce new foods in a boundaried, safe way:

“I was raised with a rule that if we ate out at a buffet I had to pick one food I'd never tried before, before I was allowed to eat whatever else I wanted; and if we ate at home, I had to eat everything that was given to me (I literally spent hours sitting with my dinner, especially if it was squash or asparagus) but I was allowed to ask for a "no-thank-you portion," which is smaller. So I feel like that habit helped me diversify my tastes and it definitely gave me courage to try new things” (Participant 9).

By contrast, one participant felt that taking control of their own eating, separate to the habits they had been raised with as part of their family, was key to improving their eating habits and their attitude towards food. For this participant, self-education as an adult around diet and nutrition was key to this process:

“I think not knowing that my body weight was actually linked to how much I put down my neck- no one ever told me that the two were linked together- was just a big issue as well. So I think this has been a lifelong thing for me. Never knowing that the two were related, never knowing it was something that I had at least some degree of control, responsibility for. As a responsible adult. I think once I discovered that about five or six years ago and once I discovered that it was something that I could change and I did have some power over, then that was empowering in itself and that’s when I started to take some control over it” (Participant 3).

#### Sub-theme: Coping as an adult

Participants commonly described one of the key ways they coped with the influence of autism on their eating as an adult as simply avoiding problem areas, and finding ways to cope around any potential difficulties. For example, one participant who ate a vegan diet due to their self-identified special interest in the environment and animals was careful to take food supplements and eat a balanced diet to ensure they received the necessary nutrition. Similarly, individuals who struggled to eat in restaurants would avoid these kind of locations, and where unavoidable would find ways to ameliorate this difficulty: for example, by asking their partner to order their food for them.

One key area of difficulty which participants described finding ways to adapt around was that of cooking. Cooking was commonly described as a difficult task, either due to executive functioning difficulties or due to sensory aversion to touching or preparing certain foods. Some participants described managing this by predominantly eating in preferred restaurants (where restaurants were not an environmental aversion), or by eating pre-prepared meals. Others used only familiar recipes which represented less of an executive functioning burden. Individuals with families described difficulties cooking as they sometimes were unable to eat the same meal as the rest of their family. One participant managed this by cooking all meals at the weekend, enabling them and their family to individually choose from a selection of meals during the week. Significantly, in the case of cooking and meal planning for the week, participants described using their tendency towards routine-based behaviour as a strength: they found that planning meals ahead of time helped both with cooking and eating, “making meal plans helps, I think, because I then kind of have to—I don’t get into a panic about “oh, what should I eat”. I’ve kind of got it all sorted out before” (Participant 11).

#### Sub-theme: Support from others

Participants also described support from family and friends as vital in managing their eating. This included having food prepared for them in a specific way, being encouraged to try new things, and being reminded to eat. Crucially, support was seen as most helpful where it respected their boundaries:

“They also know about my "nope" reflex. Some things just make me go "nope" and they don't chase me with them or ask me to try them anyway… We all abide by these boundaries and it helps tremendously” (Participant 3).

#### Sub-theme: Medical professionals

The only participants who had ever discussed their eating with a medical professional were those who had sought treatment for EDs. Of those who sought treatment, they found the experience most beneficial when the impact of their autism on their eating was acknowledged, considered and respected: “My experiences, be they atypical, are still perceived as valid” (Participant 1). However, participants felt that this required a knowledge of autism and its potential influence on eating that medical professionals may not have:

“I think it would be helpful if more professionals were aware of the sensory differences and the frequently-comorbid gastrointestinal issues that can make eating difficult for people with autism, as well as the samefooding phenomenon and other routines. Also, in general, they need to understand that meltdowns and sensory overload aren't tantrums or bad behaviour and can't be helped by treating them as such—especially with food, it's not as simple as being picky, it can range from unpleasant to uncomfortable to painful” (Participant 9).

Participants both with and without EDs strongly felt that medical professionals needed to view the influence of autism on their eating as intrinsic, rather than as a choice. They also emphasised that their eating should not be unnecessarily pathologized or seen as an illness:

“I think that would be the biggest thing I would want medical professionals to know, not necessarily for myself but for my youngest daughter. To know that there is a category of people who have these eating issues and they don’t actually have anything to do with the shape of their body. And to tell them that they need to change is wrong. It’s ethically wrong and it’s going to ethically fail, it’s going to cause these people more damage. It’s not the best way to go about things” (Participant 3).

Where the individual was experiencing eating problems, participants felt that the best way to approach this would be using flexibility, and having knowledge of the difficulties commonly associated with autism and eating, whilst retaining awareness that different people will have different experiences.

## Discussion

To date, the majority of research on eating on the autism spectrum has focused on children [1]. The experiences of participants in this study generally suggest that their eating behaviours improved from childhood into adulthood, resonating with previous research suggesting improvements throughout childhood [6]. Participants indicated they now ate a broader range of foods, and experienced less distress around eating, to the extent that they did not feel that their eating behaviours were problematic or represented a particular difficulty. However, the influence of autism on eating was felt to persist into adulthood: contributing factors documented in this study included sensory sensitivity [27, 28], persisting routine-based behaviours, and aversion to new foods. The key difference between childhood difficulties and adulthood differences suggested by this study was that participants had actively worked to cope with these core traits, either by deliberately challenging themselves (e.g. to try new foods), or through adaptation (e.g. avoiding sensory-aversive foods). This reflects research that the adaptive skills of autistic people improve into adulthood, particularly in the domain of daily living [37].

However, adulthood did appear to raise new, life-stage specific difficulties surrounding eating. As participants in this study moved out of the childhood family environment and became more independent, this raised new problems- most notably, with cooking. Executive functioning difficulties are common in autism, and this study suggests that this may make cooking a difficult, off-putting task for some autistic people [32]. Ways of coping described in this study included using meal plans, becoming familiar and confident with specific recipes, using pre-prepared meals, and asking family to prepare food. The findings of this study suggest that some autistic adults may benefit from practical support with cooking- although there is a lack of research in this area, there is a suggestion that cooking skills may improve with structured instructions and information [38, 39]. A related issue raised by this study is that outside the structured environment of family mealtimes, some participants reported forgetting to eat. This was also linked to difficulties with interoception- specifically, detecting hunger or satiety, which is an area which is known to be atypical in autism [40]. Again, autistic people who experience these difficulties may benefit from structured meal plans to avoid reliance on hunger and satiety cues.

A strong finding from this study was that participants felt that their eating behaviours should not be unnecessarily pathologized: whilst they viewed autism as impacting their eating, participants had generally found ways to cope with this impact. For the majority of participants in this study, they viewed their eating habits as different, not problematic, and wanted medical professionals to sensitively acknowledge this difference rather than unnecessarily challenging their behaviours. Interventions or outside assistance was seen as most helpful where it helped participants adapt around their behaviours, or gradually explore them in a boundaried way, rather than aiming for fundamental change. However, in this wider context, half of participants had experienced problematic eating in the form of EDs. That participants in this study viewed their autism as contributing to their EDs resonates with previous research. There is a well-documented relationship between autism and AN: individuals with AN are more likely to be on the autistic spectrum compared to the general population [34, 41]. That this study suggests that autism may influence eating behaviours, and contribute to restriction, is in line with research on co-occurring autism and AN: a recent study interviewing autistic people with AN found that they strongly viewed autism as contributing to the disordered eating behaviours through the associated cognitive rigidity, sensory sensitivity, and executive functioning[42]. However, there is less research on autism and other EDs, particularly BED. That autism was perceived by participants in this study to be related to overeating suggests that there is a potential need for more research on autism and its relation to disordered eating behaviours beyond restriction.

The need to explore the relationship between autism and eating behaviours beyond restriction is emphasised by the finding that the majority of participants in this sample had a BMI defined in the overweight range. There was a divergence in this study between the perception of the majority of autistic participants, who viewed their eating behaviours as having no significant negative effects on their health, and the fact that the majority of these participants also had a weight defined in an unhealthy range. Weight problems are not unique to autistic people: the World Health Organisation (WHO) estimates that 39% of adults are overweight, and 13% are obese [43]. However, research does suggest that both adults and children on the autism spectrum may be at higher risk of being overweight, with one study finding that as many as 34% of autistic adults may have weights in the obese range [33]. This suggests that autism may be linked to eating behaviours that lead to excessive energy intake [22, 44, 45]. The results of the present study suggest that the cognitive rigidity and routine-based behaviours associated with autism could contribute towards these eating behaviours: for example a compulsion towards completing meals, restricting one’s diet to high calorie foods only, or having routines around repeatedly eating high calorie foods. Furthermore, atypical interoception could also add to these behaviours by making it difficult to identify satiety, leading to overeating [40].

However, the issues raised in this present study may indicate that traditional medical approaches to managing weight, typically aimed at neurotypical people, may not be as effective for autistic people. Participants in this study commonly viewed the aspects of autism that contributed towards their eating behaviours, such as sensory sensitivity, as intrinsic and unchangeable. These aspects may hinder traditional weight loss approaches: for example, attempts to limit caloric intake may be limited if the individual’s diet is restricted to high calorie foods. Research into weight management programmes for children and youths on the autistic spectrum suggest that successful interventions involve individualisation, including tailoring of any dietary or behavioural recommendations to consider aspects such as sensory sensitivity [46]. However, further research is required for the adult population and in consultation with adults.

### Limitations

This was a qualitative study intended to explore eating behaviours with autistic adults. Consequently, this study used a small sample size, and further research is required to establish whether these findings are generalisable to the wider autistic population. It is possible the recruitment approach- advertising the initial online study as exploring problematic eating in autism- may have led to a self-selecting sample already interested in autism and its potential contribution to eating problems. The final study sample also had a majority of female participants, whereas autism is a condition which predominantly affects men by a ratio of around 3:1 [47]. This could additionally limit generalisability to the wider autistic population. Furthermore, participant characteristics- including weight, autism diagnosis, and ED diagnosis, were all self-reported.

Notably, all participants interviewed in this study were able to verbally communicate, and the eating behaviours and difficulties raised by this population may be qualitatively different to other autistic populations, such as those with intellectual disabilities. For example, no participants in this sample reported pica (the compulsive and repetitive consumption of inedible or non-nutritive items), despite pica being observed in autistic individuals with intellectual disabilities [48]. A further limitation of this study was that, whilst focusing on eating behaviours, it did not explore the closely related issue of exercise and physical activity. Whilst the findings of this study give insight in the potential eating behaviours that may contribute towards excessive energy intake in autistic populations, research suggests that this may be exacerbated by autistic people being less physically active [49]. Further research into the heightened risk of being overweight in autistic people should explore exercise as a potential factor, as well as the eating behaviours raised in this current study.

### Future research and clinical recommendations

Whilst the majority of participants in this study viewed the impact of their autism on their eating as a difference, some participants did feel that traits associated with their autism contributed towards difficulties with eating, including the development of EDs. That autistic adults may experience difficulties with their eating related to their autism is a topic currently under-explored in the research literature, and requires further attention. The majority of studies in this small research area have focused on the relationship between autism and restrictive eating in AN [34]; whereas the findings of this study suggest that traits associated with autism (e.g. cognitive rigidity and repetitive behaviour) can also contribute to over-eating and weight gain.

In particular, there is a need for further research on the implications of these findings for clinical practice. Where autistic adult presents to medical services with eating difficulties or problems with weight management, the experiences of participants in this study strongly suggest that clinicians need to explore the potential role of autism in these issues. This could include sensory sensitivity, routinic behaviour, or difficulties with executive functioning. At present, there is one self-report questionnaire aimed at assessing these kind of eating and mealtime difficulties in autistic adults: the SWedish Eating Assessment for Autism spectrum disorders (SWEAA) [31]. The SWEAA yields information on a number of areas, including interoceptive problems, difficulties with social eating, issues with physical coordination, and disordered eating behaviours. More research is required to understand how this kind of information could be incorporated into specialised dietary interventions for this population. Alternatively, a key finding from this current study was that some autistic adults experience issues like sensory sensitivity or routinic behaviour as a difference, rather than a difficulty. Therefore, although clinicians may identify the presence of these kind of behaviours, they will likely need to explore with the autistic individual to what extent these represent difficulties that require treatment, or whether the behaviour should be accepted by the clinician as a difference which the person is content to live with.
